# Electroconvulsive therapy induces remodeling of hippocampal co-activation with the default mode network in patients with depression

**DOI:** 10.1016/j.nicl.2023.103404

**Published:** 2023-04-12

**Authors:** Niklaus Denier, Sebastian Walther, Sigrid Breit, Nicolas Mertse, Andrea Federspiel, Agnes Meyer, Leila M. Soravia, Meret Wallimann, Roland Wiest, Tobias Bracht

**Affiliations:** a Translational Research Center, University Hospital of Psychiatry and Psychotherapy, University of Bern, Bern, Switzerland; b Translational Imaging Center (TIC), Swiss Institute for Translational and Entrepreneurial Medicine, Bern, Switzerland; c Institute of Diagnostic and Interventional Neuroradiology, University of Bern, Bern, Switzerland

**Keywords:** ECT, White matter, Resting state functional connectivity, Hippocampus, Uncinate fasciculus, parahippocampal cingulum, posterior cingulate cortex

## Abstract

Electroconvulsive therapy (ECT) is a highly efficient treatment for depression. Previous studies repeatedly reported an ECT-induced volume increase in the hippocampi. We assume that this also affects extended hippocampal networks. This study aims to investigate the structural and functional interplay between hippocampi, hippocampal pathways and core regions of the default mode network (DMN). Twenty patients with a current depressive episode receiving ECT-treatment and twenty age and sex matched healthy controls (HC) were included in the study. ECT-patients underwent multimodal magnetic resonance imaging (MRI)-scans (diffusion weighted imaging, resting state functional MRI) before and after an ECT-index series. HC were also scanned twice in a similar between-scan time-interval. Parahippocampal cingulum (PHC) and uncinate fasciculus (UF) were reconstructed for each participant using manual tractography. Fractional anisotropy (FA) was averaged across tracts. Furthermore, we investigated seed-based functional connectivity (FC) from bilateral hippocampi and from the PCC, a core region of the DMN. At baseline, FA in PHC and UF did not differ between groups. There was no baseline group difference of hippocampal-FC. PCC-FC was decreased in ECT-patients. ECT induced a decrease in FA in the left PHC in the ECT group. No longitudinal changes of FA were found in the UF. Furthermore, there was a decrease in hippocampal-PCC-FC, an increase in hippocampal-supplementary motor area-FC, and an increase in PCC-FC in the ECT-group, reversing group differences at baseline. Our findings suggest that ECT induces structural and functional remodeling of a hippocampal-DMN. Those changes may contribute to ECT-induced clinical response in patients with depression.

## Highlights

• ECT modifies coupling between the hippocampus and the default mode network.
• ECT induces microstructural alterations in the parahippocampal cingulum.
• ECT reverses reductions in posterior cingulate cortex functional connectivity (FC)
• ECT decreases FC between the hippocampus and the posterior cingulate cortex.
• ECT increases FC between the hippocampus and the supplementary motor area.

## 1 Introduction

Depression is a highly prevalent disorder with an estimated lifetime prevalence of 16 % (Kessler et al., 2003). It is the leading cause for lost years due to disability (WHO, 2021). Pharmacotherapy and psychotherapy are effective treatments for depression. However, about one third of patients with depression remains treatment resistant and does not respond to multiple pharmacological treatment regimens (Rush et al., 2006). Out of these patients, about 50 % respond to electroconvulsive therapy (ECT) (Group, 2003, van Diermen et al., 2018). This has led to an increasing interest in the neurobiological underpinnings of ECT-treatment response.

ECT-induces volume increases in the hippocampi (Gryglewski et al., 2019, Gryglewski et al., 2021, Nuninga et al., 2020, Oltedal et al., 2018, Takamiya et al., 2018), which is also the case for the sample of this study (Bracht et al., 2023). Given that structural and functional alterations of the hippocampus have been linked to the pathophysiology of depression (Bracht et al., 2022a, Bremner et al., 2000, Sheline et al., 2019), researchers assumed that hippocampal alterations may be a mechanism of ECT-treatment response (Nordanskog et al., 2010). However, large mega- and *meta*-analysis failed to show an association between reductions of depression severity and hippocampal volume increase (Gryglewski et al., 2021, Oltedal et al., 2018). It is possible, that more subtle changes in hippocampal subfields (e.g. the dentate gyrus) or spatially distributed networks related to anterior or posterior segments of the hippocampi may underlie treatment response (Bracht et al., 2023, Leaver et al., 2021, Nuninga et al., 2020). Investigations of extended hippocampal networks may therefore contribute to elucidate the role of the hippocampus in ECT-treatment response.

The hippocampus is a hub exhibiting structural covariance with multiple brain regions, including the default mode network (DMN) (Kotkowski et al., 2018). The DMN is a large-scale brain network that is primarily composed of posterior cingulate cortex (PCC), precuneus, medial prefrontal cortex (mPFC) and angular gyrus (Buckner et al., 2008). Functionally, the DMN is relevant for non-task related self-referential processing, which directs the attention inwards (Andrews-Hanna et al., 2010). For example, internal mentation is related to self-referential thinking including remembering the past and daydreaming (Andrews-Hanna, 2012). Negative self-generated thought (rumination), a core symptom of depression, has been linked to aberrant activity within the DMN as well (Hamilton et al., 2015). Rumination has been associated with both an overactivation of the mPFC (anterior DMN) (Berman et al., 2011, Hamilton et al., 2011, Zhu et al., 2012) and with decreased activity of the PCC (posterior DMN) (Mulders et al., 2016b). Rumination is likely influenced by the hippocampus due to its core role for consolidation of information from short-term memory to long-term memory (encoding) and for retrieval of episodic memory (Squire et al., 2015). This assumption is in line with findings of functional MRI studies demonstrating that the hippocampus is coupled with the DMN during memory retrieval (Huijbers et al., 2011). Effects of ECT in depression have been linked to an increase in functional connectivity (FC) of the PCC (Abbott et al., 2013, Leaver et al., 2016). These ECT induced connectivity changes may ameliorate aberrant mentation. Previous research points to ECT-induced alterations in FC between the hippocampus and various cortical regions (Bai et al., 2019, Leaver et al., 2021, Sinha et al., 2020, Takamiya et al., 2020). However, structural and functional connectivity between the hippocampus and functional hubs of the DMN have not been investigated specifically in ECT studies. Further investigation of remodeling of hippocampal coactivation with the DMN may help elucidate ECT treatment response in depression.

Alterations of FC between hippocampus and DMN must rely on structural connections. These connection pathways can be reconstructed indirectly with help of diffusion tensor imaging (DTI)-based fibre tracking (Jones et al., 2013). The mPFC is connected with the hippocampus through the uncinate fasciculus (UF) that connects the orbitofrontal cortex/ mPFC with the temporal lobe (Catani et al., 2002). Posterior regions of the DMN (PCC/ precuneus) form part of the parahippocampal cingulum (PHC) (Jones et al., 2013). One probabilistic fibre tracking study investigated tracts emanating from the anterior hippocampus and found alterations of diffusion properties (axial (AD), radial (RD) and mean diffusivity (MD) that were specific for ECT-responders (Kubicki et al., 2019). Another study used a hippocampal region of interest (ROI)-approach and identified ECT-induced decreases in fractional anisotropy (FA) in the hippocampus, which were not correlated with clinical response (Jorgensen et al., 2016).

This study aims to investigate ECT-induced alterations in functional and structural connectivity of brain regions and pathways linking the hippocampus with the DMN. We hypothesized the following alterations of structural and functional connectivity in ECT-patients at baseline compared to healthy controls: First, increased FC between the hippocampus and regions of the DMN, which may reflect an increased tendency for rumination (Huijbers et al., 2011). Second, decreased baseline PCC-FC (Mulders et al., 2016b). Third, alterations of structural connectivity in the uncinate fasciculus and the PHC as assessed with FA. Fourth, we hypothesized an ECT-induced normalization of those measures of FC and structural connectivity during the course of an ECT-index series.

## 2 Methods

### 2.1 Participants

Our study sample includes participants of previous cross-sectional (Bracht et al., 2022a, Bracht et al., 2022b, Mertse et al., 2022) and longitudinal studies (Bracht et al., 2023). Patients with a current depressive episode that were scheduled for an ECT-index series at the ECT-clinic of the University Hospital of Psychiatry and Psychotherapy Bern were asked if they were interested to participate. Inclusion criteria for patients were age between 18 and 65 years, a diagnosis of major depressive disorder (MDD) or bipolar disorder (BD) according to the Diagnostic and Statistical Manual of Mental Disorders (DSM-5), American Psychiatric Association (APA, 2013), and a clinically informed decision to enrol in an ECT-index series. Exclusion criteria were neurological disorders, addictive disorders, psychotic disorders, personality disorders, known claustrophobia or other contraindications to perform an MRI-scan. Patients with symptoms of anxiety disorders were not excluded given the high comorbidity and symptom overlap with depression. Diagnostic screening was performed using the Mini International Neuropsychiatric Interview (MINI) (Sheehan et al., 1998) and the Structured Clinical Interview for DSM-IV Axis II (SCID-II) (Wittchen et al., 1997). We used the Edinburgh Handedness Inventory (Oldfield, 1971) for the assessment of handedness. The 21-item Hamilton Rating Scale for Depression (HAMD) (Hamilton, 1967) and the 21-item Beck Depression Inventory-II (BDI-II) (Beck et al., 1996) were used for the assessment of depression severity. Depression rating scales were administered before and after an ECT-index series at the day of the MRI-scan. Twenty healthy controls matched for age and sex underwent an identical assessment. Written informed consent was obtained from all subjects and the local cantonal ethics committee approved the study (KEK-number: 2017-00731).

### 2.2 ECT treatment

ECT-treatment took place at the recovery room of the University Hospital in Bern (Inselspital), Switzerland. A Thymatron IV system was used. Most patients (n = 17) were treated with right unilateral stimulation. Out of these 17 patients, five patients switched to bitemporal stimulation and one patient switched to bifrontal stimulation during the ECT-index series. Two patients received an ECT-index series with bitemporal stimulation, and one patient was treated with bifrontal stimulation. Decisions on initial electrode positioning and switches during the ECT-index series were made based on the clinical presentation and course. We used the titration-based method to determine initial seizure threshold and stimulus intensity. ECT-patients had an average of 12.7 ± 4.0 ECT-sessions between MRI-scans. General anesthesia was performed using etomidate; succinylcholine was used for muscle relaxation. Seizure quality was monitored with help of electroencephalogram and electromyography recordings.

### 2.3 MRI data acquisition

Each participant was scanned at two timepoints with a 3 Tesla MRI scanner (Magnetom Prisma, Siemens, Erlangen, Germany) equipped with a 64-channel head and neck coil at the University Hospital of Bern. The ECT-group was scanned before and after an ECT-index series. The healthy control group was also scanned at two timepoints with a similar inter-scan duration to the ECT-group (see Table 1). For acquisition of T1-weighted data, we used a bias-field corrected MP2RAGE sequence with acquisition of two gradient volumes (INV1 and INV2) and a high-contrast volume (UNI). The following parameters were used for acquisition of MP2RAGE: FOV = 256 × 256, matrix = 256 × 256, slices = 256, voxel resolution = 1 × 1 × 1 mm^3^, TR/TE = 5000/2.98 ms, TI = 700 ms and T2 = 2500 ms. A spin-echo echo-planar sequence was used for acquisition of diffusion-weighted imaging (DWI). The following parameters were used: 64 × b1000 (1000 s/mm^2^), 1 × b0 (0 s/mm^2^), 60 Slices, FOV = 269 × 269, 128 × 128 matrix, 2.2 × 2.2 × 2.2 mm^3^ isotropic resolution, TR = 6200 ms, TE = 69 ms. Echo planar imaging (*EPI*) was used for continuous acquisition of an 8 min lasting resting-state functional MRI with ‘eyes closed’ condition. The following parameters were used: 480 volumes with 48 slices per volume, FOV = 230 × 230, 94 × 94 matrix, 2.4 × 2.4 × 2.4 mm^3^ isotropic resolution, TR = 1000 ms, and TE = 30 ms.

**Table 1:** Demographics for all patients and healthy controls.

	ECT (n = 20)		Controls (n = 20)		P Value
Age (years)	44.9 ± 12		43.6 ± 14		0.75
Sex (female, male)	8, 12		8, 12		1.00
Handedness (right, left, ambidextrous)	15, 2, 3		17, 2, 1		0.57
BDI-II (Baseline)	30.6 ± 8.1		1.5 ± 2.4		**<0.001**
HAMD-21 (Baseline)	21.4 ± 5.3		0.65 ± 1.0		**<0.001**
Duration of episode (months)	19.8 ± 17		n.a.		n.a.
Number of episodes	5.2 ± 4		n.a.		n.a.
Time between scans (days)	52.6 ± 24		61.2 ± 17		0.20
BDI-II (Follow up)	20.8 ± 10.3		1 ± 1.8		**<0.001**
HAMD-21 (Follow up)	10.9 ± 8.1		0.25 ± 0.8		**<0.001**
n (remitter^1^)	9		n.a.		n.a.
n (responder or remitter^2^)	11		n.a.		n.a.
n (non-responder)	9		n.a.		n.a.
Medication	T1	T2	T1	T2	

SSRI (%)	20	15	0	0	n.a.
Dual antidepressants (%)	55	50	0	0	n.a.
Tricyclic antidepressants (%)	15	20	0	0	n.a.
Tranylcypromine (%)	5	10	0	0	n.a.
Moclobemide (%)	5	5	0	0	n.a.
No antidepressant (%)	10	5	100	100	n.a.
Lithium (%)	35	30	0	0	n.a.

Demographic and clinical variables were compared between ECT and healthy controls using an independent t-tests and $\chi^{2}$ tests. Abbreviations: ECT: electroconvulsive therapy; HC: healthy controls; ^1^ remitter: HAMD-21 < 8 points; ^2^ responder or remitter: HAMD-21 reduction > 50 %; n.a.: not applicable.

### 2.4 Diffusion weighted imaging and manual tractography

**Fig. 1:**
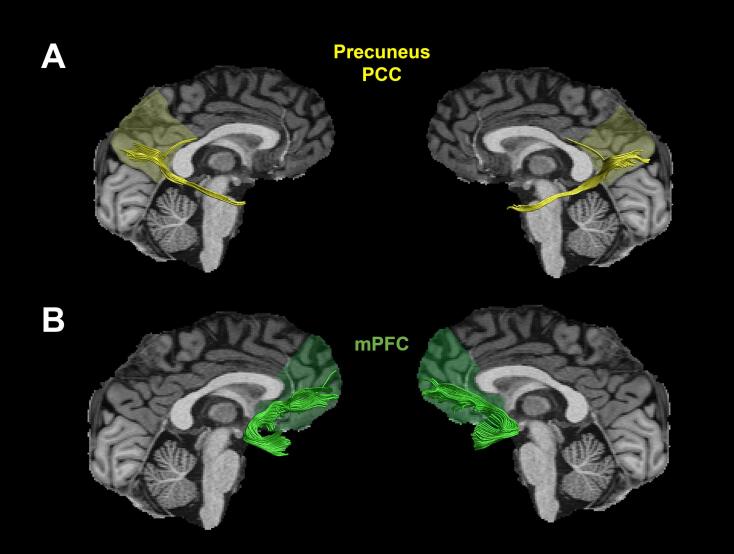
Example of structural connectivity of the hippocampus to the DMN for both hemispheres. **A:** Reconstruction of the parahippocampal cingulum (PHC) and its projection to the posterior cingulate cortex (PCC). **B:** Reconstruction of the uncinate fasciculus (UF) and its projection to the medial prefrontal cortex (mPFC).

We used FSL 6.0 (https://www.fmrib.ox.ac.uk/fsl/) and FSL-BET for robust brain extraction (-R option). Because of noisy background of MP2RAGE UNI images, we performed a brain extraction using INV2-images as input and applying the derived binary mask to the UNI-image. DWI scans were analyzed using ExploreDTI 4.8.6 (Leemans et al., 2009). A correction for subject motion was performed by co-registering the DWI-images to the b0-image (Leemans and Jones, 2009). An echo planar imaging (*EPI*) correction for eddy current distortions and field inhomogeneities was executed warping the motion corrected DWI-images to the brain extracted MP2RAGE image (Wu et al., 2008). DTI based whole-brain deterministic tractography was performed applying a diffusion tensor model (Basser et al., 1994). Tractography termination criteria were FA < 0.2 and angle threshold > 45 degrees. ROI delineation was performed using manual tractography (see Fig. 1). The UF was reconstructed on coronal sections. Two ROIs were drawn approximately at the height of the NAcc surrounding the temporal lobe and the region lateral to NAcc and putamen (Bracht et al., 2015). The PHC was reconstructed as proposed by Jones et al. (Jones et al., 2013): Two “AND-ROIs” were positioned on horizontal sections using colour coded first eigenvector-fractional anisotropy (FEFA) images. One ROI was placed at the most ventral section to the splenium of the corpus callosum. The second ROI was positioned four slices above. Five slices posterior to the rostro-caudal midpoint of the body of the corpus callosum a “NOT-gate” was positioned on a coronal section (“restricted PHC”) (Jones et al., 2013). Spurious fibres that do not correspond to known anatomy were eliminated using further “NOT-gates”. Finally, mean FA, MD, RD, and AD were extracted from reconstructed bilateral PHC and UF tracts.

### 2.5 Resting-state functional MRI

We analysed resting-state fMRI using the CONN 21a toolbox (Whitfield-Gabrieli and Nieto-Castanon, 2012). Pre-processing steps included realignment and co-registration of *EPI* volumes to MP2RAGE, segmentation and normalisation to the MNI space, and smoothing using an FWHM kernel of 8 × 8 × 8 mm. We applied band-pass filtering (0.008–0.09 Hz) to remove physiological signals and regress nuisance variables of each of the five timeseries within segmented white matter and cerebrospinal fluid and 12 realignment parameters. Scrubbing of outlier volumes with global BOLD signal or framewise displacement (FD) higher than the 97th percentile was performed using the Artefact Detection Tools (ART) toolbox implemented in CONN. Additionally, for every subject, we computed the mean FD. Seed-based FC of the PCC and the hippocampus was computed for each timepoint (baseline and follow up) and each participant.

### 2.6 Statistics

The Statistical Package for Social Sciences SPSS 28.0 (SPSS, Inc., Chicago, Illinois) was used for data analyses. Two-sample t-tests were used for comparison of dimensional demographic variables between groups. $\chi^{2}$-tests were applied to compare categorical variables.

#### 2.6.1 Analyses of diffusion MRI data

Baseline group comparisons between the ECT-group an HC was performed using separate ANCOVAs for the UF and the PHC with the independent variable group (ECT, HC), the within subject factor hemisphere, the dependent variable FA and the covariates age and sex. To investigate if time effects between the 2 MRI-scans differ between ECT-patients and HC, two separate repeated measures ANCOVAs with the independent variable group (ECT, HC), the within subject factors timepoint and hemisphere, the covariates age and sex and the dependent variable FA of the UF and the PHC were calculated. Significant group × time effects were followed up using post hoc paired t-tests comparing FA between baseline and follow up for each group. Significant results for FA were investigated with further paired t-tests comparing MD, RD and AD between baseline and follow up.

#### 2.6.2 Analyses of functional MRI data

We performed baseline between-group analyses and group × time interactions using a full-factorial model. Motion parameters were regressed in first-level analyses. Second-level analyses were performed with seed-based FC of the bilateral hippocampus and PCC (AAL atlas) and were corrected with regressors age, sex and mean FD. We used a voxel threshold of p < 0.01 and a cluster correction with a false discovery rate (FDR) correction of p < 0.05. Significant clusters in baseline differences and group × time interactions are displayed in Fig. 3 and in Table 3, Table 4 including information on anatomical area, hemisphere, cluster size and standardised MNI (Montreal Neurological Institute and Hospital) coordinates.

#### 2.6.3 Associations with between structural and functional neuroplasticity and clinical response

For imaging measures with significant group × time interaction and significant differences from baseline to follow up in the ECT group we calculated exploratory correlations between the difference of the imaging variables (follow up – baseline) and the overall clinical improvement (HAMD follow up – baseline) (see supplementary material S1 and S2). Furthermore, we complement our main analyses and show contrasts between follow-up measures between groups and between baseline and follow up measure for the ECT group regardless of significant group × time interactions (supplementary material S3-S5).

## 3 Results

Twenty-five ECT-patients completed the baseline assessment. Five of these patients were lost during follow-up (claustrophobia during MRI-scanning (n = 1), data acquisition error (n = 1), study withdrawal on patients request (n = 2), COVID-19 associated (n = 1). Thus, the final sample of our analysis consisted of 20 ECT-patients. Sixteen patients had a diagnosis of MDD and four patients had a diagnosis BD. All ECT-patients were treatment resistant to antidepressive medication or did not tolerate antidepressants with adequate dosage. Seven patients met criteria for double-depression (mean duration 15.1 ± 8.6 years). Two patients had a history of a previous ECT-treatment. Due to the observational design of our study, patients were not required to be on stable medication and medication was changed during the ECT-treatment. Fifty-five per cent of ECT-patients achieved remission or response. ECT and HC groups did not differ regarding age, sex, handedness and time between scans (for information that is more detailed see Table 1).

### 3.1 Comparison of UF and PHC structural connectivity

**Fig. 2:**
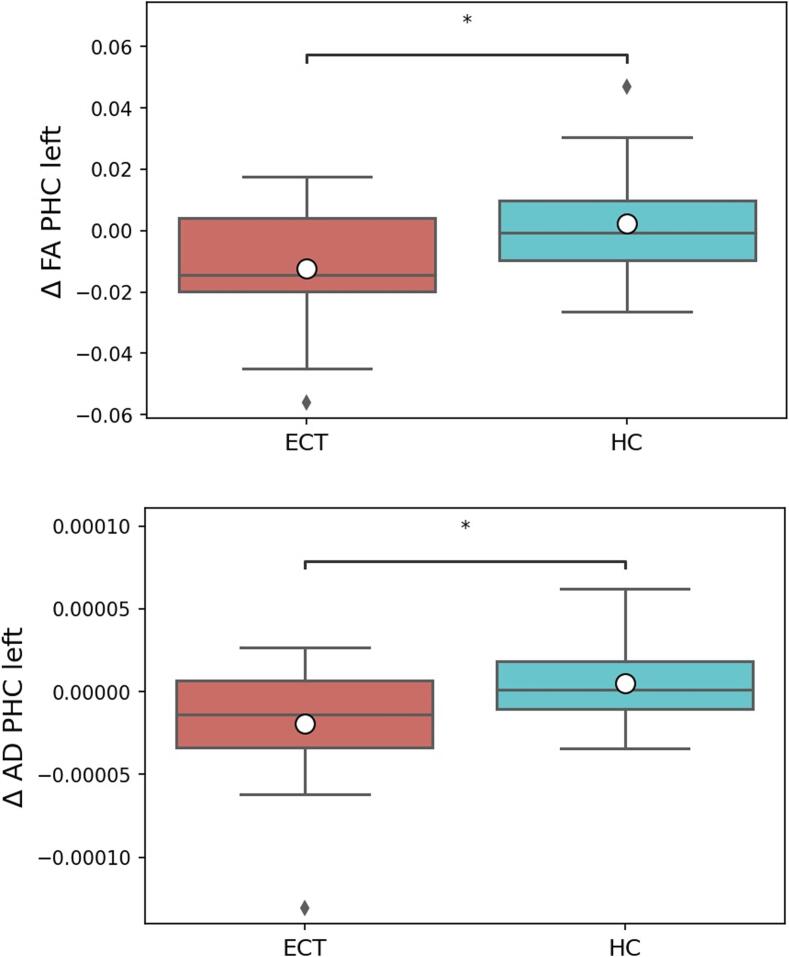
Group differences in structural connectivity of the left PHC measured by fractional anisotropy (FA) and axial diffusivity (AD).

Baseline comparisons did not show any differences in FA for the PHC (F (1, 36) = 0.692, p = 0.411) or the UF (F (1, 36) = 1.555, p = 0.220). Longitudinal analyses revealed a significant group × time interaction for FA of the PHC (F (1, 36) = 4.335, p < 0.045, η^2^ = 0.107), but not for the uncinate fasciculus (F (1, 36) = 0.924, p = 0.343). Follow up paired t-tests for the PHC revealed a decrease of FA and AD in the left PHC in the ECT-group (see Table 2 and Fig. 2).

**Table 2:** Post hoc t-tests for white matter microstructure of the parahippocampal cingulum (PHC) between patients and healthy controls.

	ECT	HC
Post hoc paired t-tests for FA of left and right PHC		
FA (left)	T (19) = 2.971, **p = 0.008**	T (19) = -0.602, p = 0.554
FA (right)	T (19) = 1.903, p = 0.072	T (19) = 1.018, p = 0.321

Follow up paired t-tests for further diffusion properties of the left PHC		
MD (left)	T (19) = 0.871, p = 0.395	n.a.
RD (left)	T (19) = -1.233, p = 0.233	n.a.
AD (left)	T (19) = 2.429, **p = 0.025**	n.a.

Abbreviations: FA; fractional anisotropy; MD: mean diffusivity; RD: radial; AD: axial.

### 3.2 Comparison of functional connectivity

**Fig. 3:**
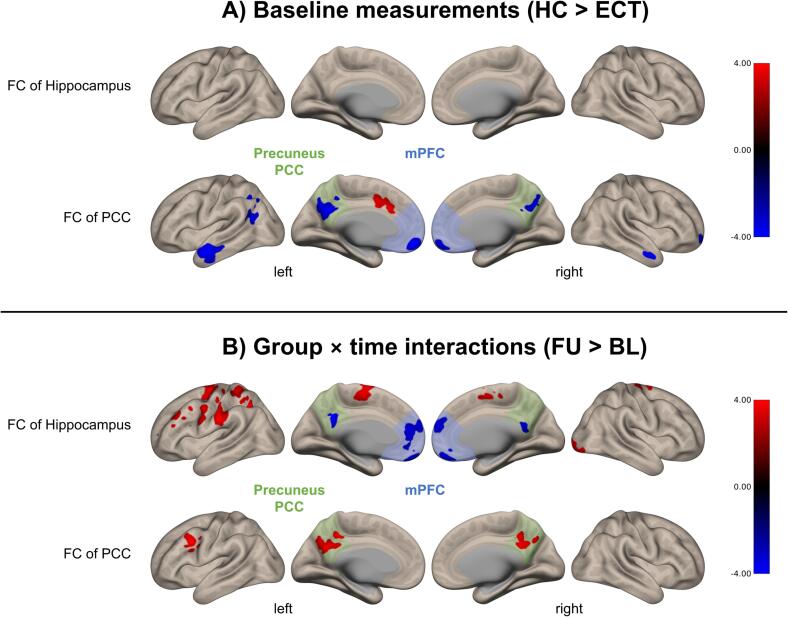
Group differences (ECT vs HC) and group × time interaction in seed-based functional connectivity of the hippocampus and the posterior cingulate cortex **A:** Group differences in baseline condition. Red colour indicates HC > ECT and blue colour HC < ECT. **B:** Group × time interaction of contrast ECT > HC and follow up > baseline. Red colour indicates ECT > HC / follow up > baseline and blue colour ECT > HC / follow up < baseline. Only clusters with an FDR < 0.05 correction are displayed. (For interpretation of the references to colour in this figure legend, the reader is referred to the web version of this article.)

Analysing seed-based FC of bilateral hippocampi we found no significant group differences for the baseline condition (see Table 3 and Fig. 3A). However, there was a significant group × time interaction. We identified reductions of FC between the hippocampi and bilateral mPFC and PCC in the ECT group. In contrast, ECT-patients showed increased FC between the hippocampi and bilateral precentral gyri and supplementary area (SMA), left postcentral gyrus, left superior parietal lobule and left middle frontal gyrus (see Table 3 and Fig. 3B).

**Table 3:** Seed based functional connectivity of the hippocampus.

Group Differences	ECT Group	Area	Hemisphere	Cluster (voxels)	MNI (x y z)	p-FDR
BL: ECT vs HC	None.					

Longitudinal Effects	**Effect FU**	**Area**	**Hemisphere**	**Cluster (voxels)**	**MNI** **(x y z)**	**p-FDR**

Group × time ECT vs HC	↑	Precentral gyrus / SMA	B	734	−6–8 78	0.00001
	↓	mPFC	B	477	0 62 22	0.00035
	↑	Postcentral gyrus	L	375	−60–20 28	0.0015
	↑	Occipital pole	R	335	26–102 −4	0.0025
	↓	mPFC	B	280	6 52–20	0.0063
	↑	Precentral gyrus	L	248	−50 4 44	0.01
	↑	Superior parietal lobule	L	211	–32–54 40	0.02
	↑	Postcentral gyrus	L	206	–22–38 56	0.02

Abbreviations: SMA: supplementary motor area; mPFC: medial prefrontal cortex; PCC: posterior cingulate cortex; B: both hemispheres; L: left hemisphere; R: right hemisphere.

In the analysis of seed-based FC of the PCC, there were significant baseline group differences with reduced FC in patients with the bilateral precuneus/PCC complex, left middle temporal gyrus, bilateral mPFC, left angular gyrus and left superior parietal lobule. Increased baseline PCC-FC was found in the ECT group in the right amygdala and left accumbens and left anterior cingulate gyrus (see Table 4 and Fig. 3A). Investigating group × time interactions we found increased PCC-FC within the bilateral PCC and the left middle frontal gyrus in the ECT-group (see Table 4 and Fig. 3B).

**Table 4:** Seed based functional connectivity of the posterior cingulate cortex (PCC).

Group Differences	ECT Group	Area	Hemisphere	Cluster (voxels)	MNI (x y z)	p-FDR
BL: ECT vs HC	↓	Precuneus / PCC	B	629	−2–64 32	0.00009
	↓	Middle temporal gyrus	L	602	−56–4 –22	0.00009
	↓	mPFC	B	333	−6 54–14	0.005
	↓	Middle temporal gyrus	R	285	62 2–30	0.01
	↑	Amygdala	R	262	16 4–14	0.01
	↑	Accumbens	L	220	−16 6–12	0.02
	↓	Angular gyrus	L	203	−46–60 26	0.03
	↓	Superior parietal lobule	L	188	–32–54 42	0.03
	↑	Anterior cingulate gyrus	L	186	−8 18 30	0.03

Longitudinal Effects	**Effect FU**	**Area**	**Hemisphere**	**Cluster (voxels)**	**MNI** **(x y z)**	**p-FDR**

Group × time ECT vs HC	↑	Precuneus / PCC	B	654	−2–44 42	0.000045
	↑	Middle frontal gyrus	L	240	−42 6 36	0.038

Abbreviations: PCC, posterior cingulate cortex; mPFC, medial prefrontal cortex (mPFC); ECT, electroconvulsive therapy; HC, healthy controls; B: both hemispheres; L: left hemisphere; R: right hemisphere; BL: baseline; FU: follow up.

## 4 Discussion

This study combines analysis of DTI with resting state fMRI to investigate ECT-induced alterations in structural and functional connectivity between the hippocampus and midline regions of the DMN in patients with a current depressive episode. We identified an ECT-induced reduction of FA in the left PHC pointing to remodeling of this pathway, which connects the hippocampus with the PCC. Alterations of structural connectivity were accompanied by a decrease of FC between the hippocampus and the mPFC/ PCC and an increase in FC between the hippocampus and the SMA in the ECT-group. This points to an extended hippocampal network that is modified by ECT-treatment. Additionally, we found a normalization of PCC-DMN FC after ECT, which may point to intrinsic remodeling within the DMN.

After an ECT-index series, we found a decrease of FA in the left PHC, which connects the hippocampus with the PCC. Follow-up analyses found decreases of AD but not of MD and RD. Our finding adds to rather diverse findings of alterations of ECT-induced findings of diffusion properties (Kubicki et al., 2019, Lyden et al., 2014, Repple et al., 2020). Reductions in AD in the absence of changes in RD point to axonal remodelling (Sun et al., 2006). Pre-translational electroconvulsive stimulation (ECS) research suggests that ECT-induces neurogenesis in the hippocampi (Madsen et al., 2000, Olesen et al., 2017). Furthermore, ECS results point to an increase in hippocampal dendritic arborization, cell proliferation and synaptogenesis (Jonckheere et al., 2018, Sartorius et al., 2022). Such processes of neuronal remodeling may increase the architectural complexity of the PHC consequently leading to the observed reductions of FA and AD. In contrast, no alterations of diffusion properties were found in the UF. Therefore, structural plasticity may be specific for the PHC. Assuming that changes in FA and AD in the PHC are driven by neuroplasticity of the hippocampus it is also possible that tractography lacks sensitivity to capture such effects in the UF, which is a larger fibre bundle with strong connections to further brain regions (e.g. amygdala, OFC) that may not undergo remodeling during ECT.

Alterations of the PHC, which projects to the PCC, may influence further networks. We found reduced FC between the PCC and the mPFC in patients with depression at baseline, which is in line with previous studies investigating rumination (Satyshur et al., 2018). Previous findings suggest that PCC-mPFC FC is positively correlated with reflective rumination which is conceptualized as a purposeful and solution-oriented way of ruminating (Satyshur et al., 2018). Thus, decreased PCC and PCC-mPFC FC within the DMN as identified in our study in the ECT group likely reflects a dysfunction of reflective rumination. Our results point to recovery of PCC and PCC-mPFC FC during ECT, which is in line with previous studies reporting ECT-induced increases of FC in the DMN (Mulders et al., 2016, Pang et al., 2022). One may speculate that recovery of PCC and PCC-mPFC FC within the DMN may underlie the shift from brooding rumination to a more reflective thinking pattern, which is typically observed during the process of recovery in depression (Roberts et al., 2021).

Functional coupling between the hippocampus and core regions of the DMN may be linked to rumination in depression due to the role of the hippocampus for memory retrieval (Huijbers et al., 2011). We identified an ECT-induced decrease of hippocampal-PCC and hippocampal-mPFC FC, while ECT induced FC increases between the hippocampus and the SMA. While decreases of hippocampal-PCC and hippocampal-mPFC FC may reflect a decrease of rumination, increases in FC between the hippocampus and the SMA may be associated with normalization of lack of drive, which is commonly observed during the process of remission (Bracht et al., 2015). However, specific rating scales for rumination and assessments of psychomotor behaviour (e.g. actigraphy measures (Bracht et al., 2014)) would be required to further address the behavioural implications of observed changes in FC.

Finally, our study has some limitations. First, sample size is small. Second, depressed patients are heterogeneous regarding diagnoses (unipolar and bipolar depression) and clinical characteristics (e.g. severity and duration of episode, medication). Third, we cannot rule out an impact of medication changes. However, this reflects everyday clinical practice and enhances generalizability (external validity). Fourth, DTI-based assessments are unspecific regarding neurobiological compartments (e.g. axonal properties, myelination) and do not allow conclusions on the type of structural remodeling. Finally, longitudinal studies need to test whether the changes observed directly after the ECT series will be maintained or lost during follow-up.

In sum, our data suggests ECT-induced remodeling of functional and structural connectivity between the hippocampus and core midline regions of the DMN. Our results suggest that ECT induces plasticity of hippocampal networks that go beyond the frequently reported ECT-induced volumetric changes of the hippocampi (Bracht et al., 2023, Ousdal et al., 2020). Future studies should extend our findings and link measures of structural and functional connectivity of the hippocampus to specific clinical syndromes associated with the DMN (e.g. measures of rumination).

## Role of the funding source

This work was supported by the Robert Enke Foundation (to Tobias Bracht and Sebastian Walther), by the Novartis Foundation for Medical Biological Research (#19C203) (to Tobias Bracht and Sebastian Walther) by the Swiss Life Foundation (to Tobias Bracht), and by the Adrian et Simone Frutiger Foundation (to Tobias Bracht).

## CRediT authorship contribution statement

**Niklaus Denier:** Conceptualization, Data curation, Formal analysis, Methodology, Supervision, Writing – original draft, Writing – review & editing. **Sebastian Walther:** Conceptualization, Funding acquisition, Writing – review & editing. **Sigrid Breit:** Data curation, Writing – review & editing. **Nicolas Mertse:** Data curation, Writing – review & editing. **Andrea Federspiel:** Resources, Software, Supervision, Writing – review & editing. **Agnes Meyer:** Data curation, Writing – review & editing. **Leila M. Soravia:** Conceptualization, Formal analysis, Supervision, Writing – review & editing. **Meret Wallimann:** Data curation, Formal analysis, Writing – review & editing. **Roland Wiest:** Resources, Software, Supervision, Writing – review & editing. **Tobias Bracht:** Funding acquisition, Conceptualization, Data curation, Formal analysis, Methodology, Supervision, Writing – original draft, Writing – review & editing.

## Declaration of Competing Interest

The authors declare that they have no known competing financial interests or personal relationships that could have appeared to influence the work reported in this paper.

## Data Availability

Data will be made available on request.
