# Supplementary material for: Electroconvulsive therapy induces remodeling of hippocampal co-activation with the default mode network in patients with depression
Source: Neuroimage Clin. 2023 Apr 12;38:103404. doi: 10.1016/j.nicl.2023.103404 (PMC10130338; doi:10.1016/j.nicl.2023.103404)
Supplement: Supplementary data 1 [file mmc1.docx]

**S1:** Intermodal Spearman correlations between significant DTI und functional connectivity (FC) measurements.


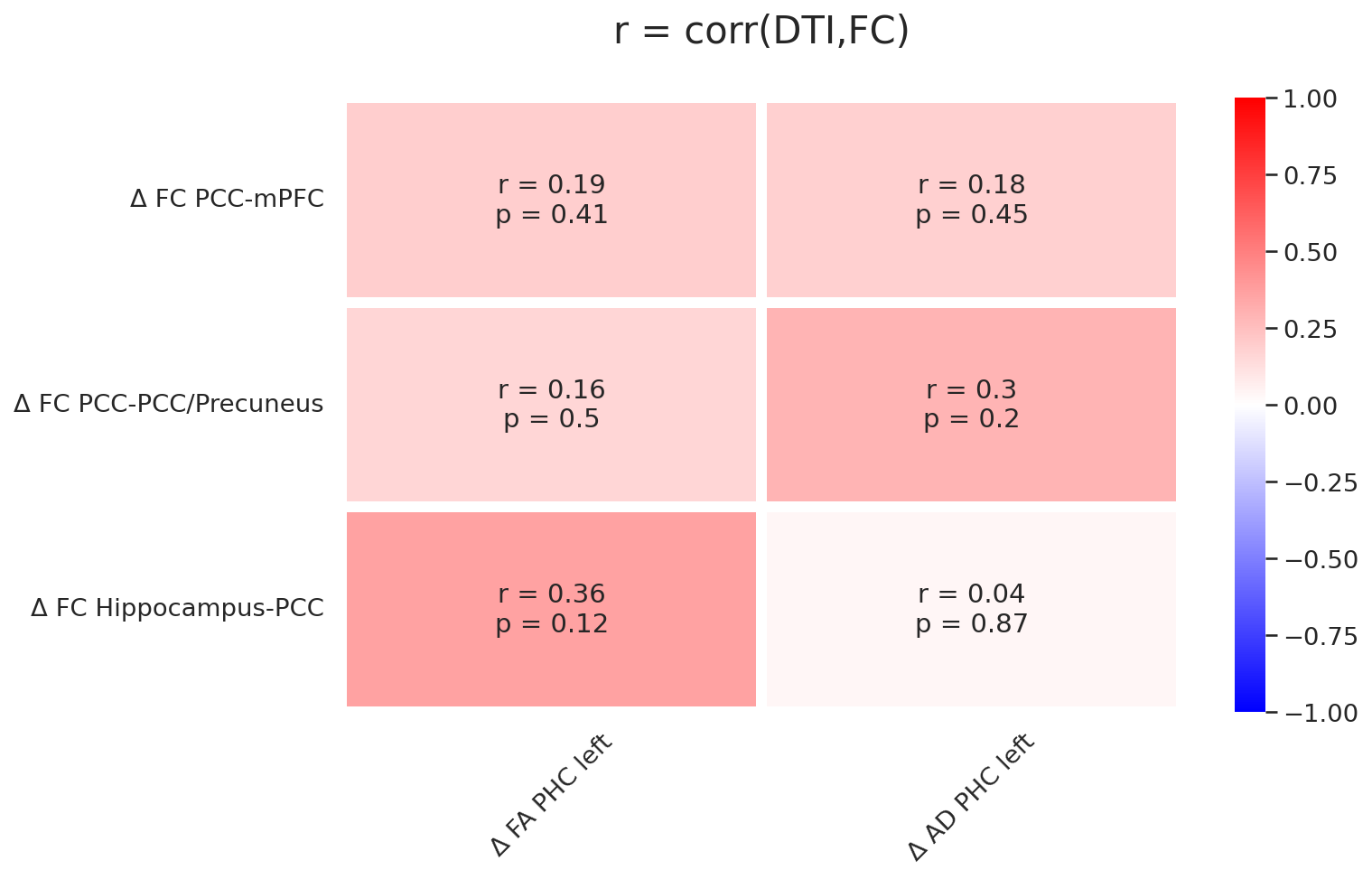


Abbreviations: PCC, posterior cingulate cortex; mPFC, medial prefrontal cortex; PHC: parahippocampal cingulum; FA: fractional anisotropy; AD: axial diffusivity; FC: functional connectivity.

**S2:** Intermodal Spearman correlations between HAMD reductions and significant structural and functional connectivity results.


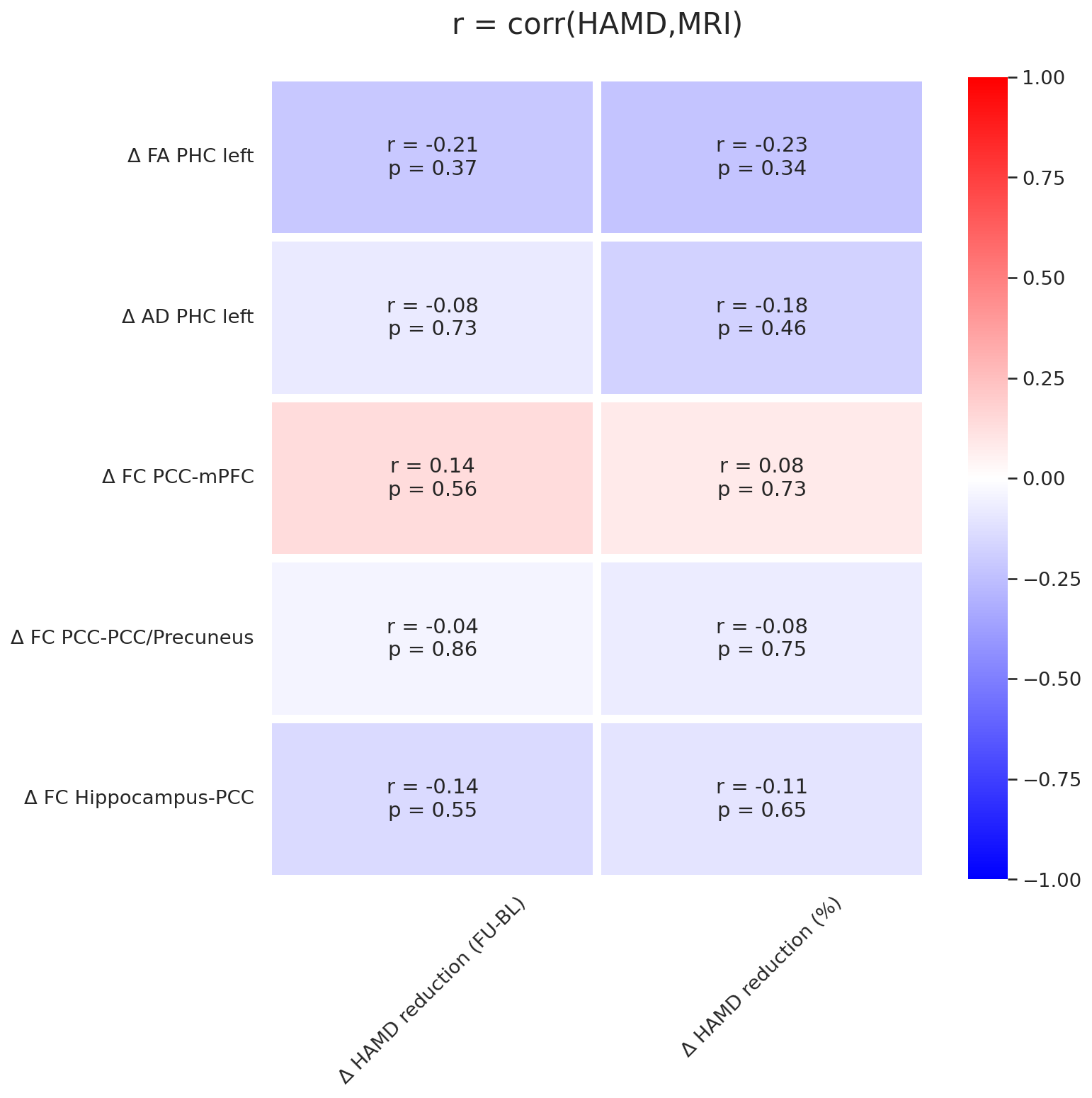


Abbreviations: HAMD: Hamilton Depression Rating Scale; PCC, posterior cingulate cortex; mPFC, medial prefrontal cortex; PHC: parahippocampal cingulum; FA: fractional anisotropy; AD: axial diffusivity; FC: functional connectivity.

**S3:** Seed based functional connectivity of the hippocampus.

| Group  Differences | ECT Group | Area | Hemisphere | Cluster (voxels) | MNI  (x y z) | p-FDR |
| --- | --- | --- | --- | --- | --- | --- |
| FU: ECT vs. HC | None |  |  |  |  |  |
| Longitudinal  Effects | **Effect FU** | **Area** | **Hemisphere** | **Cluster (voxels)** | **MNI**  **(x y z)** | **p-FDR** |
| ECT (BL vs. FU) | ↓ | Paracingulate gyrus | B | 507 | 2 60 22 | 0.0003 |
|  | ↑ | Superior frontal gyrus | B | 458 | -10 -10 62 | 0.0004 |
|  | ↓ | mPFC | B | 318 | 6 52 -20 | 0.004 |
|  | ↓ | PCC / Precuneus | B | 275 | -12 -50 28 | 0.007 |
|  | ↑ | Postcentral gyrus | L | 188 | -60 -20 28 | 0.001 |
|  | ↑ | Superior frontal gyrus | R | 172 | 20 -06 76 | 0.05 |

**Group Differences:** Results of independent T-tests controlling for age and sex.
**Longitudinal Effects:** Results of paired t-Tests for the ECT-group controlling for age and sex.

**Abbreviations:** BL: baseline; FU: follow up; mPFC: medial prefrontal cortex; PCC: posterior cingulate cortex; ECT: electroconvulsive therapy; HC: healthy controls; B: both hemispheres; L: left hemisphere; R: right hemisphere.

**S4:** Seed based functional connectivity of the posterior cingulate cortex (PCC).

| Group  Differences | ECT Group | Area | Hemisphere | Cluster (voxels) | MNI  (x y z) | p-FDR |
| --- | --- | --- | --- | --- | --- | --- |
| FU: ECT vs. HC | ↓ | Cuneal cortex | B | 509 | -2 -88 26 | 0.0002 |
| Longitudinal  Effects | **Effect FU** | **Area** | **Hemisphere** | **Cluster (voxels)** | **MNI**  **(x y z)** | **p-FDR** |
| ECT: BL vs. FU | ↑ | Precuneus / PCC | B | 681 | -2 -44 42 | 0.00003 |
|  | ↑ | Middle frontal gyrus | L | 241 | -40 6 36 | 0.04 |

**Group Differences:** Results of independent T-tests controlling for age and sex.
**Longitudinal Effects:** Results of paired t-Tests for the ECT-group controlling for age and sex.

**Abbreviations:** BL: baseline; FU: follow up; PCC, posterior cingulate cortex; mPFC, medial prefrontal cortex (mPFC); ECT: electroconvulsive therapy; HC: healthy controls; B: both hemispheres; L: left hemisphere; R: right hemisphere.

**S5:** DTI analyses of parahippocampal cingulum (PHC) and uncinate fasciculus (UF)

| Group  Differences | Tract | Result |
| --- | --- | --- |
| FU: ECT vs. HC | PHC | F( 1, 36) =0.53, p = 0.47 |
| FU: ECT vs. HC | UF | F( 1, 36) =0.60, p = 0.44 |
| Longitudinal  Effects | **Tract** | **Result** |
| ECT: BL vs. FU | PHC (left) | P = 0.008 |
| ECT: BL vs. FU | PHC (right) | P = 0.072 |
| ECT: BL vs. FU | UF (left) | P = 0.159 |
| ECT: BL vs. FU | UF (right) | P = 0.362 |

**Group Differences:** Results of repeated measures ANCOVA with dependent variable group, independent variable FA, and within subject factor hemisphere, controlling for age and sex. **Longitudinal Effects:** results of paired t-tests for the ECT-group are shown.

**Abbreviations**: ECT: electroconvulsive therapy; HC: healthy controls; PHC: parahippocampal cingulum; UF: uncinate fasciculus.
